# Identification of a putative methyltransferase gene of *Babesia bigemina* as a novel molecular biomarker uniquely expressed in parasite tick stages

**DOI:** 10.1186/s13071-018-3052-9

**Published:** 2018-08-24

**Authors:** Gamila A. R. Bohaliga, Wendell C. Johnson, Naomi S. Taus, Hala E. Hussein, Reginaldo G. Bastos, Carlos E. Suarez, Roberta O’Connor, Massaro W. Ueti

**Affiliations:** 1 Program in Vector-borne Diseases, Department of Veterinary Microbiology and Pathology, Washington State University, Pullman, Washington, 99164 USA; 2 Animal Diseases Research Unit, USDA-ARS, Pullman, Washington, 99164-6630 USA; 3 Department of Entomology, Faculty of Science, Cairo University, Giza, 12613 Egypt; 4 The Paul G. Allen School for Global Animal Health, Washington State University, Pullman, Washington, 99164-70403 USA

**Keywords:** *Babesia bigemina*, Sexual stages, *In vitro* induction, TCEP, Parasite-specific tick stage genes, *Rhipicephalus microplus*

## Abstract

**Background:**

Bovine babesiosis is caused by apicomplexan pathogens of the genus *Babesia* such as *B. bigemina* and *B. bovis*. These tick-borne pathogens have a complex life-cycle involving asexual multiplication in vertebrate hosts and sexual reproduction in invertebrate vectors. In the tick midgut, extracellular *Babesia* parasites transform into gametes that fuse to form zygotes. Understanding the mechanisms that underlie formation of extracellular *Babesia* tick stages is an important step towards developing control strategies for preventing tick infection and subsequent parasite transmission.

**Results:**

We induced *B. bigemina* sexual stages *in vitro* by exposing parasites to Tris 2-carboxyethyl phosphine (TCEP). Subsequently, we identified a novel putative methyltransferase gene (BBBOND_0204030) that is expressed uniquely in all *B. bigemina* tick stages but not in blood stages. *In vitro* TCEP-exposed *B. bigemina* presented diverse morphology including parasites with long projections, round forms and clusters of round forms indicative of sexual stage induction. We confirmed the development of sexual stages by detecting upregulation of previously defined *B. bigemina* sexual stage marker genes, *ccp2* and *3*, and their respective protein expression in TCEP-induced *B. bigemina* cultures. Next, transcription analysis of *in vitro* TCEP-induced *B. bigemina* culture based on an *in silico* derived list of homologs of *Plasmodium falciparum* gamete-specific genes demonstrated differential expression of the gene BBBOND_0204030 in induced cells. Further examination of *ex vivo* infected ticks demonstrated that BBBOND_0204030 is transcribed by multiple stages of *B. bigemina* during parasite development in tick midgut, ovary and hemolymph. Interestingly, *ex vivo* results confirmed our *in vitro* observation that blood stages of *B. bigemina* do not express BBBOND_0204030 and validated the *in vitro* system of inducing sexual stages.

**Conclusions:**

Herein we describe the identification of a *B. bigemina* gene transcribed exclusively by parasites infecting ticks using a novel method of inducing *B. bigemina* sexual stages *in vitro*. We propose that this gene can be used as a marker for parasite development within the tick vector. Together, these tools will facilitate our understanding of parasite-tick interactions, the identification of novel vaccine targets and, consequently, the development of additional strategies to control bovine babesiosis.

**Electronic supplementary material:**

The online version of this article (10.1186/s13071-018-3052-9) contains supplementary material, which is available to authorized users.

## Background

Cattle tick fever is a globally distributed bovine apicomplexan disease that is transmitted by multiple ixodid tick species. *Babesia bigemina* and *B. bovis* are the main causative agents of cattle tick fever in tropical and subtropical regions. This disease has a negative economic impact on the cattle industry due to the cost of treatment, morbidity and mortality. Animals infected with *B. bigemina* develop high fever, lethargy, severe anemia, and hemoglobinuria. Animals that survive become persistently infected for life and are reservoirs for tick transmission [1]. *Babesia bigemina* is more prevalent than other *Babesia* parasites because it is transmitted by several tick species including *Rhipicephalus microplus*, *R. annulatus*, *R. appendiculatus*, *R. evertsi*, *Boophilus decoloratus* and *B. geigyi*, as well as multiple stages of these ticks, including nymph and adult stages [1–5]. Similar to *Plasmodium* spp., the causative agent of malaria, *B. bigemina* has a complex life-cycle, including asexual reproduction in the mammalian host and sexual reproduction in the tick host. The *B. bigemina* life-cycle in the mammalian host begins when infected nymphs or adults of the tick vector feed on a bovine. Tick vectors inoculate sporozoites during the blood meal and parasites invade erythrocytes. Within erythrocytes, parasites transform into trophozoites, and then merozoites which lyse the infected cells and invade new erythrocytes, perpetuating the cycle of asexual reproduction in the bovine host. Adult female ticks ingest *B. bigemina* blood stages during feeding on an infected animal. In the tick midgut, parasite sexual stages (gametes) are induced which fuse to form zygotes that invade midgut epithelial cells. *Babesia* kinetes are released into the hemolymph and transmitted to tick progeny, in what is known as transovarial transmission [1, 6]. There remain fundamental knowledge gaps regarding the role of *B. bigemina* tick stage genes involved in parasite development within the arthropod vector.

The availability of arthropod-borne apicomplexan parasite genomes provides the opportunity to identify genes that contribute to parasite perpetuation within mammalian and arthropod vector hosts [7–10]. Those available genomes have facilitated the identification of genes and gene families that are conserved across the genera *Plasmodium* and *Babesia*, such as the cysteine motif-rich gene family, the *ccp* gene family and the *hap2* gene. These conserved genes are expressed by *Plasmodium* spp. and *Babesia* spp. gametes during parasite development within the biological vectors [11–17]. In malaria, *in vitro* approaches have been used to test efficacy of drugs or vaccines to block parasite transmission [18]. One of the challenges in studying *Babesia* gametocytes is the lack of *in vitro* techniques to consistently induce gametogenesis which restricts evaluation of drugs or vaccines targeting parasite sexual stage proteins. Two studies have successfully induced *B. bigemina* sexual stages using xanthurenic acid, but both studies required *ex vivo* blood stages as starting materials [13, 19]. However, malaria gametogenesis can be induced from *in vitro* adapted parasites. This has been accomplished by using a reducing agent, dithiothreitol (DTT) which induces endoplasmic reticular stress resulting in morphological and transcriptional transformations leading to gametogenesis [20, 21]. Similar to DTT, Tris 2-carboxyethyl phosphine (TCEP) is a reductant [22].

A study of four stages of *P. falciparum* including sporozoites, merozoites, trophozoites, and gametocytes revealed that there are specific genes uniquely expressed by each stage in the life-cycle of malaria parasites [23]. Like *Plasmodium*, *Babesia* genes are expected to be expressed at specific life-cycle stages during infection of the mammalian host or tick vector. In this study, we describe the identification of a novel tick-stage specific protein encoded by gene BBBOND_0204030 using bioinformatic approaches and TCEP to induce *B. bigemina* sexual stages *in vitro*. Importantly, gene BBBOND_0204030 was expressed by *in vitro* induced *B. bigemina* sexual stages and in multiple *in vivo* parasite tick stages but not by blood stages.

## Methods

### *In vitro* induction of *B. bigemina* sexual stages

Culture adapted *B. bigemina* was used for inducing sexual stages *in vitro*. In brief, *B. bigemina-*infected erythrocytes were grown in HL-1 medium (Lonza, Walkersville, MD, USA) supplemented with 40% normal bovine serum, 10 mM 3-[N-tris (hydroxymethyl) methylanino]-2-hydroxypropanesulfonic acid (Sigma-Aldrich, St. Louis, MO, USA), and antibiotic/antimitotic (Sigma-Aldrich), pH 7.2, with 5% packed cell volume of bovine red blood cells. To increase parasitemia, cultures were incubated at 37 °C in 5% CO_2_ and expanded without addition of erythrocytes as previously described [24]. When the parasitemia exceeded 10%, *B. bigemina* sexual stages were induced by adding either TCEP (Thermo Fisher Scientific, Grand Island, NY, USA) or DTT (Thermo Fisher Scientific, Fair Lawn, NJ, USA) [20, 21]. TCEP and DTT stock concentrations were at 500 mM for both reagents. The final concentration of reducing agents in induction medium was 0, 10, 20, 25, 30 or 40 mM and contained equal volumes of solvent. Induction medium was balanced to pH 7.2 by the addition of 5 M HCl. Cultures were incubated for 1 h at 26 or 37 °C with or without 5% CO_2_. Cultures were then washed once with fresh medium to remove excess of reducing agent and centrifuged at 2655× *g* for 3 min. Parasites were cultured in fresh medium with or without 100 μM xanthurenic acid (Sigma-Aldrich) and incubated for up to 24 h at 26 or 37 °C with or without 5% CO_2_. Smears were made from all cultures at 3, 6, 15 and 24 h post-induction and stained with Hema 3 stain (Thermo Fisher Scientific, Waltham, MA, USA) to analyze the formation of sexual stages.

To estimate cell viability, *B. bigemina* non-induced and TCEP-induced cultures were incubated in two different condition at 37 °C with or without 5% CO_2_ for 24 h. Parasites were collected and washed 3 times with 1× phosphate buffered saline (PBS), suspended in 1× PBS and mixed with equal volume of 6-carboxyfluorescein diacetate (6-CFDA) [25] final concentration in 1× PBS 20 μg/ml (Calbiochem-Behring, La Jolla, CA, USA). Cells were incubated at room temperature for 15 min and washed once with 1× PBS. Cells pellets were suspended in 1× PBS and one drop of ProLong Gold anti-fade reagent with DAPI (Invitrogen, Eugene, OR, USA) was added to each sample under a coverslip. All samples were visualized under a Leica microscope (Buffalo Grove, IL, USA) using LAS-X software.

### Scanning electron microscopy

Morphology of *Babesia bigemina* sexual stages was analyzed for greater detail using scanning electron microscopy. In brief, *B. bigemina-*induced cultures were centrifuged at 400× *g* for 1 min to differentially pellet erythrocytes. Supernatant was recovered into new tubes and extra-erythrocytic *B. bigemina* sexual stages collected by centrifugation at 3000× *g* for 15 min. Sexual stage parasite pellets were washed three times in 1× PBS. Samples were fixed in 2% paraformaldehyde, 2% glutaraldehyde, 0.1 M phosphate buffer and incubated overnight at 4 °C. Samples were rinsed twice with distilled water and post-fixed overnight in 2% osmium tetroxide. After water rinses, samples were dehydrated with an ethanol series (30–100%). Final drying used hexamethyldisilazane (HMDS). Samples were suspended in HMDS, pipetted onto a coverslip attached to an aluminum scanning electron microscopy (SEM) stub and placed in a vacuum desiccator overnight prior to gold coating [26]. Samples were imaged on an FEI SEM Quanta 200F at the Franceschi Microscopy and Imaging Center, Washington State University, Pullman, WA, USA.

### Reverse transcriptase real-time quantitative PCR (RT-qPCR) of *B. bigemina* sexual stage marker genes

To test the expression level of defined sexual stage *B. bigemina ccp2 (*BBBOND_0203950) and *ccp3 (*BBBOND_0312400) genes in cultures induced with TCEP, RT-qPCR was performed [12]. Non-induced and TCEP-induced parasite cultures were collected at 0, 6, 15 and 24 h post-induction and washed once with 1× PBS at 2655× *g* for 15 min, and the pellets suspended in Trizol (Thermo Fisher Scientific, Waltham, MA, USA) for total RNA isolation. Total RNA was isolated and treated with DNase-Free (Thermo Fisher Scientific, Waltham, MA, USA). Five-hundred nanograms of total RNA was utilized for cDNA synthesis using a Superscript IІI™ cDNA Synthesis Kit (Thermo Fisher Scientific, Waltham, MA, USA) following the manufacturer’s protocol. Primer sets for *B. bigemina ccp2-3* and actin were designed based on *B. bigemina* genomic sequences and listed in Table 1 [27]. The RT-qPCR was performed in a CFX96™ Real-Time PCR Detection System and reactions were performed in triplicate in 20 μl using 10 μl SsoFast™ EvaGreen® Supermix (Bio-Rad, Hercules, CA, USA), 1 μl of 500 nM of each primer set, 6 μl of nuclease free water and 2 μl of a 1:10 dilution of cDNA as template. The cycling conditions consisted of an enzyme activation step of 95 °C for 30 s followed by 40 cycles of 95 °C denaturation for 5 s and annealing/extension at 55 °C for 5 s. The *B. bigemina* actin gene (BBBOND_0107357) was used as a reference gene and the transcription level of *ccp* genes was calculated relative to time zero (non-induced) parasites, using the formula: relative expression _(sample)_ = 2 ^[C*q* (control) – C*q* (sample)]^, where time zero was used as Cq control. We used Bio-Rad CFX Manager Software version 3.1 to analyze the RT-qPCR data. Amplification efficiency and melt curve analyses were performed to evaluate analytical sensitivity and specificity of the RT-qPCR for each gene of interest. Standard PCR for *B. bigemina* actin (BBBOND_0107357) was performed to demonstrate the presence of amplifiable cDNA in all samples tested.

**Table 1:** Primer sets of *B. bigemina* homologs of *P. falciparum* gamete-specific genes

Gene	Forward primer (5'-3')	Reverse primer (5'-3')	Amplicon size (bp)
BBBOND_0204030	GAGGAAATCGATGGGACGTATG	GGTACTCCATGTGACGTTTAGG	496
BBBOND_0204030 (nested PCR)	ACATCTCTGCGTCTTGCATAA	GGACTGTATGTATGCCCTTCAG	204
BBBOND_0310420	GAGCAGGTGGCGACATATT	CCTTGATACCGTTTCCCATAGAG	330
BBBOND_0110620	GTCGAGAAAGGCCAGGATAAA	TGTGTCATGGGAGGGTTTG	382
BBBOND_0308930	CGAGGGCGCTACTCTATTC	CACTGATGTTTCGTGGCTTAC	267
BBBOND_0200260	ATGTCGAAGGATTCCACTGC	CTTGCGAGAGGATGGTGTG	208
BBBOND_0311670	GAAGCCTGAGTGGAACCTAAA	GCGGAGGTGGGATTCATATT	343
BBBOND_0208380	TCGTCGCGTGGAATTCATAG	TGTTCACCGCCTCGAAATAG	449
BBBOND_0305250	GCGAGTACTGCGGCATTTA	CTGAAGCCAAGCTGAGGTG	295
BBBOND_0307580	CGAAGATCCTCGTGCTGATAAT	GAGCGGGTTGTATAGGTACTTG	386
BBBOND_0310760	TGCTTCACCTGCGGCAA	TCAGATGTGCTCCTGGTTCC	204
BBBOND_0311270	ATCTGCAAGTTCTGGGAGAAG	CATGTAGCCCTGCTGTTGATA	477
BBBOND_0203950 (CCp2)	TCCACAACAAGCAACTCC	GCTTTGTAGGTAGAGTCAGC	168
BBBOND_0312400 (CCp3)	GAGTCCTCCGTGTAGATGAAC	GAAGCTTACTTGCTACGACAAC	141
BBBOND_0307410 (HAP2)	GCTGAAGACGAAGGTGAGGA	GCGATGATCTGGGTGACG	164
BBBOND_0107357 (actin)	ATCGCCGTTTACACTTCACG	GCCCCTTCCTCCTCGTAATC	131

### Fixed immunofluorescence assay of *B. bigemina* sexual stage maker proteins

To determine *B. bigemina* sexual stage formation, fixed immunofluorescence assays (IFA) were performed as previously described [12, 28]. In brief, non-induced and induced *B. bigemina* cultures were collected and pelleted at 3000× *g* for 15 min at 4 °C. Cells were washed twice with 1× PBS at 3000× *g* for 15 min at 4 °C and pellets suspended in 3% BSA in 1× PBS. These cells were used to make smears on glass slides which were fixed in cold acetone for 1 min. The slides were blocked with 1× PBS containing 10% goat normal serum (PBS-NGS) and incubated for 30 min at 37 °C in a humidity chamber. Slides were rinsed with ddH_2_O and air dried. In a previous study, we generated and characterized anti-CCp2 and anti-CCp3 primary antisera [12]. The antibodies were diluted 1:20 in PBS-NGS and applied to individual wells. Slides were placed in a humidity chamber for 30 min at 37 °C. Slides were then rinsed once with ddH_2_O and washed three times for 10 min with cold 1× PBS. The slides were incubated for 30 min with goat anti-rabbit Alexa Fluor 647 conjugated secondary antibody diluted 1:1000 in PBS-NGS at 37 °C. All samples were washed twice in 1× PBS, once with ddH_2_O and air dried. Finally, one drop of ProLong Gold anti-fade reagent with DAPI (Invitrogen) was added to each sample under a coverslip. Identically produced negative controls were performed using pre-immune (PI) rabbit serum as the primary antibody. All samples were visualized under a Leica microscope using LAS-X software.

### *In silico* identification of *B. bigemina* sexual stage genes

To identify *B. bigemina* homologs of *P. falciparum* gamete-specific genes, bioinformatic analysis was performed based on amino acid identity [23, 27] using NCBI Blastp (https: //blast.ncbi.nlm.nih.gov/Blast.). Clustal omega analysis (http://www.ebi.ac.uk/Tools/msa/clustalo/) was used to evaluate the percent amino acid identity of proteins. Protein domains conserved between *B. bigemina* and *P. falciparum* homologs were determined using the Simple Modular Architecture Research Tool (http://smart.embl-heidelberg.de/).

### Reverse transcriptase PCR (RT-PCR) of *B. bigemina* homologs of *P. falciparum* gamete-specific genes

To identify qualitative changes in transcript expression profiles associated with *B. bigemina* sexual transformation, transcripts from TCEP-induced culture were compared against transcripts from non-induced cultures. At 24 h post-induction, samples were collected and washed once with 1× PBS at 2655× *g* for 15 min, and the pellets suspended in Trizol for total RNA isolation. Total RNA isolation and DNase-Free treatment were performed as described above. One-hundred nanograms of total RNA was utilized for cDNA synthesis using a Superscript IІI™ cDNA Synthesis Kit (Thermo Fisher Scientific, Waltham, MA, USA) following the manufacturer’s protocol. Synthesized cDNA was used in RT-PCR to detect *B. bigemina* transcripts. Primer sets for *B. bigemina* homologs of *P. falciparum* gamete-specific genes are listed in Table 1 and were designed based on *B. bigemina* genomic sequences [27]. RT-PCR reactions were conducted in 20 μl containing 2 μl synthesized cDNA, 1 μl of 10 μM of each primer set, 6 μl nuclease-free water and 10 μl RedTaq (Sigma-Aldrich). The amplification conditions consisted of 3 min denaturation at 95 °C, 35 repeated cycles of 30 s denaturation at 95 °C, 30 s annealing at 62 °C and 30 s extension at 72 °C, with a final 7 min extension at 72 °C. Amplicons were resolved using 1% agarose gel electrophoresis. All PCR products were sequenced to determine gene specificity. Nested PCR was used to evaluate the transcription of BBBOND_0204030 in non-induced and TCEP-induced parasite cultures. Primer sets for BBBOND_0204030 are listed in Table 1. The first run of nested PCR condition with the set of external primers was 3 min of denaturation at 95 °C, 35 repeated cycles of 30 s for denaturation at 95 °C, 30 s for annealing at 62 °C and 30 s for extension at 72 °C, with a final 7 min extension at 72 °C. The second run of nested PCR condition with the set of internal primers was 3 min of denaturation at 95 °C, 35 repeated cycles of 30 s for denaturation at 95 °C, 30 s for annealing at 57.2 °C and 30 s for extension at 72 °C, with a final 7 min extension at 72 °C. Amplicons were resolved using 2% agarose gel electrophoresis.

### *Babesia bigemina* infection of adult female *R. microplus* ticks

A splenectomized Holstein calf, four months of age and tested to be bovine babesiosis-free by nested PCR and competitive enzyme-linked immunosorbent assay, was infected with a *B. bigemina* Mexico strain isolated from a naturally infected calf in Mexico [29, 30]. The La Minita strain of *R. microplus* ticks, originated from cattle on pasture in Starr County, Texas, was used for the acquisition of *B. bigemina*. We have previously demonstrated this *R. microplus* colony efficiently acquires parasites from *B. bigemina* infected calves and form kinetes in the tick hemolymph [28]. In brief, about 40,000 *R. microplus* larvae were applied under a cloth back patch on the splenectomized calf. After 14 days, when approximately 1% of ticks had molted to adults, *B. bigemina-*infected erythrocytes were inoculated intravenously into the calf to synchronize tick acquisition feeding with an ascending *B. bigemina* parasitemia. Engorged female ticks were collected 7–9 days post-*B. bigemina* infection, rinsed in tap water, and placed into tissue culture plates in airtight containers with saturated KNO_3_ solution at 26 °C and 92% relative humidity and incubated to allow formation of *B. bigemina* sexual stages and kinetes in the tick midgut and hemolymph, respectively. Incubated female ticks were examined daily for *B. bigemina* infection as previously described [31].

### Detection of stage-specific *B. bigemina* transcripts

*Babesia bigemina-*infected ticks were identified by removing the distal leg segment of engorged female ticks, dropping exuding hemolymph onto slides and staining with Giemsa. The slides were examined by light microscopy for the presence of *B. bigemina* kinetes as previously described [31]. Infected female ticks were dissected daily from day 0 through day 6 post-incubation. Tick midgut, hemolymph and ovaries were collected in Trizol (Thermo Fisher Scientific, Waltham, MA, USA) and stored at -80 °C. Total RNA was extracted from tick tissues, treated with DNase-Free, cDNA synthesized, and RT-PCR was performed as described before. All PCR products were sequenced to confirm the origin of the amplicon.

### Statistical analysis

Bio-Rad CFX Manager Software version 3.1 was used to analyze gene expression levels in a pairwise manner. The results were expressed as the mean ± SEM. P value < 0.05 was considered statistically significant.

## Results

### *In vitro* exposure of *B. bigemina* to TCEP

**Fig. 1:**
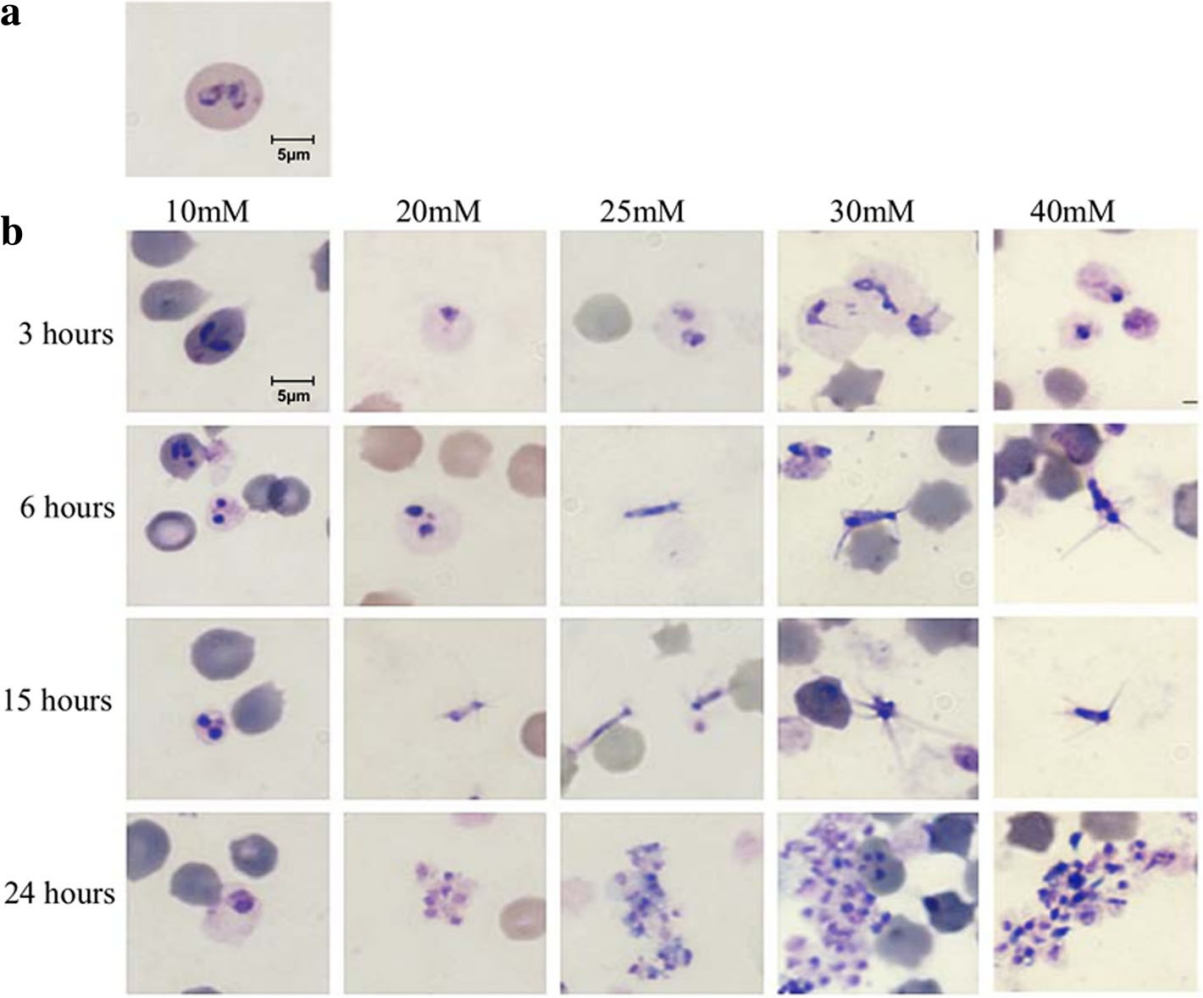
Development of *B. bigemina* sexual stages. Non-induced blood culture (time 0) (**a**) and *in vitro* TCEP-induced sexual stages (**b**) at 26 °C and 5% CO_2_ (multiple time-points). *Scale-bar*: 5 μm

**Fig. 2:**
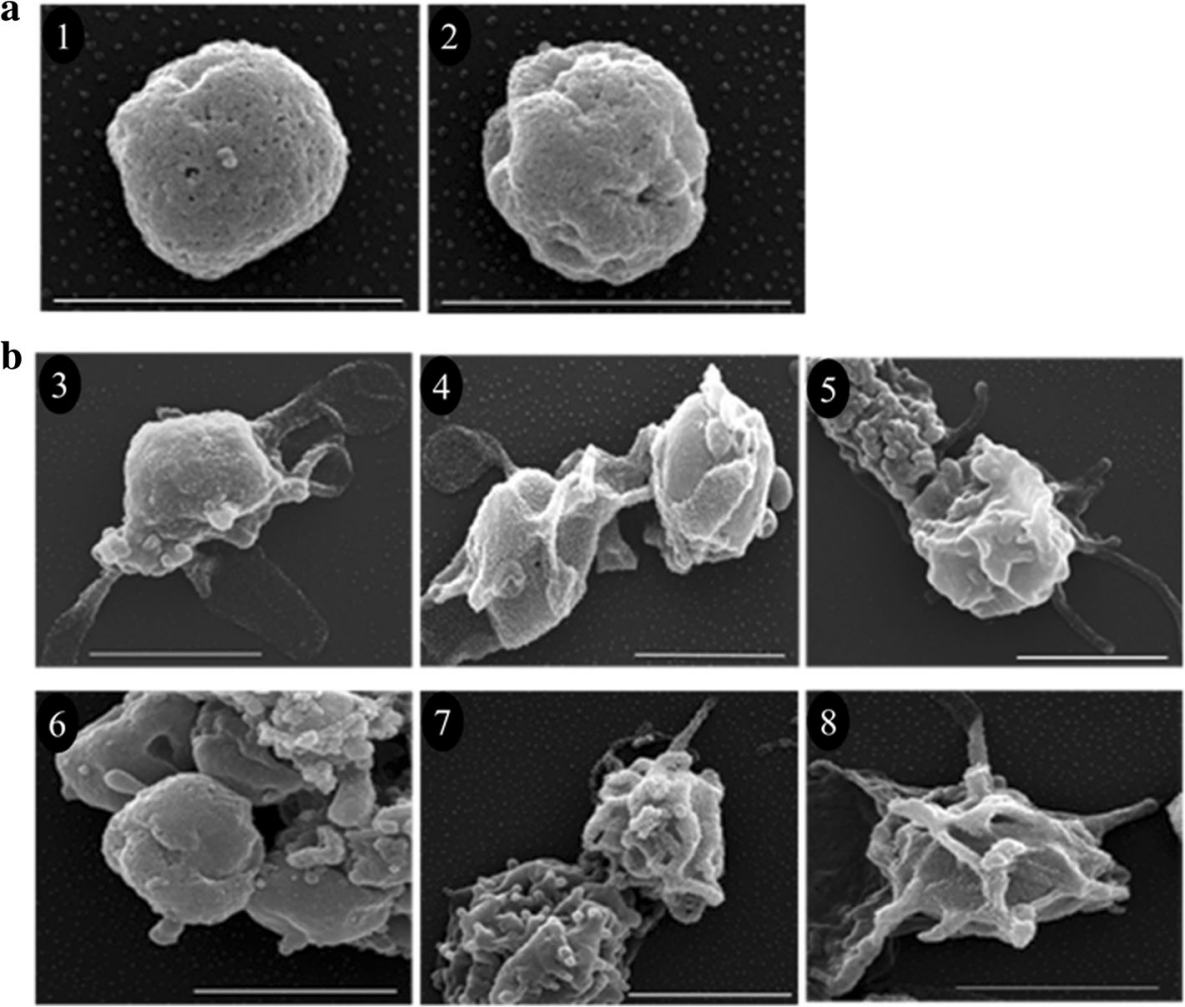
Scanning electron micrographs: **a** Non-induced blood culture (merozoite). Panels 1 and 2 show the morphologies observed. **b** *In vitro* TCEP-induced sexual stages. Panels 3–8 show different parasite morphologies observed at 24 h post-induction. *Scale-bars*: 2 μm

*Babesia bigemina* cultures were treated either with TCEP or DTT for 1 h and washed into medium with or without xanthurenic acid. Cultures were incubated at two different temperatures in the presence or absence of 5% CO_2_. Morphological changes were observed with 20, 25, 30 and 40 mM TCEP and 40 mM DTT at 26 °C and at 20 and 25 mM TCEP and 40 mM DTT at 37 °C in (Table 2) and CO_2_ was required for sexual stage formation [32]. The presence of xanthurenic acid had no effect on the induction of sexual stages. The induced parasites initially underwent morphological changes inside infected RBCs where they enlarged and occupied most of the RBC volume at 3 h. In addition to enlarged size, parasites began to make projections while still inside the erythrocytes. Parasites became extracellular at 6 and 15 h post-induction, yielding morphologically distinct populations: parasites with long projections and large, round parasite stages. The extracellular parasites began to aggregate after 24 h (Fig. 1). Staining with 6-carboxyfluorescein diacetate demonstrated the viability of induced sexual stages (Additional file 1: Figure S1). Scanning electron microscopy was performed to reveal greater detailed visualization of *B. bigemina* sexual stage morphological changes in induced parasites. Extracellular merozoites showed a round form with a size of ~1.5 μm in panels 1 and 2 (Fig. 2a) similar to a previous study [33]. Induced sexual stages had a size of ~ 2 μm. Cells with several long projections or round aggregation forms were observed (Fig. 2b). Induced aggregating forms are shown in Fig. 2b (panels 4 and 6). Parasites depicted in panels 3, 5, 7 and 8 also displayed dramatic shape and surface architecture modifications when compared to non-induced parasites [33, 34].

**Table 2:** Treatment conditions to induce *B. bigemina* sexual stages. All cultures were in the presence of 5% CO_2_. Bold indicates sexual stage induction

Condition	Inducer	Concentrations (mM)					
26 °C	TCEP	0	10	**20**	**25**	**30**	**40**
	TCEP+ XA	0	10	**20**	**25**	**30**	**40**
37 °C	TCEP	0	10	**20**	**25**	30	40
	TCEP+ XA	0	10	**20**	**25**	30	40
26 °C	DTT	0	10	20	25	30	**40**
	DTT+ XA	0	10	20	25	30	**40**
37 °C	DTT	0	10	20	25	30	**40**
	DTT+ XA	0	10	20	25	30	**40**

*Abbreviations*: TCEP, Tris 2-carboxyethyl phosphine; DTT, dithiothreitol; XA, xanthurenic acid

### Expression of *B. bigemina* sexual stage genes after *in vitro* TCEP exposure

**Fig. 3:**
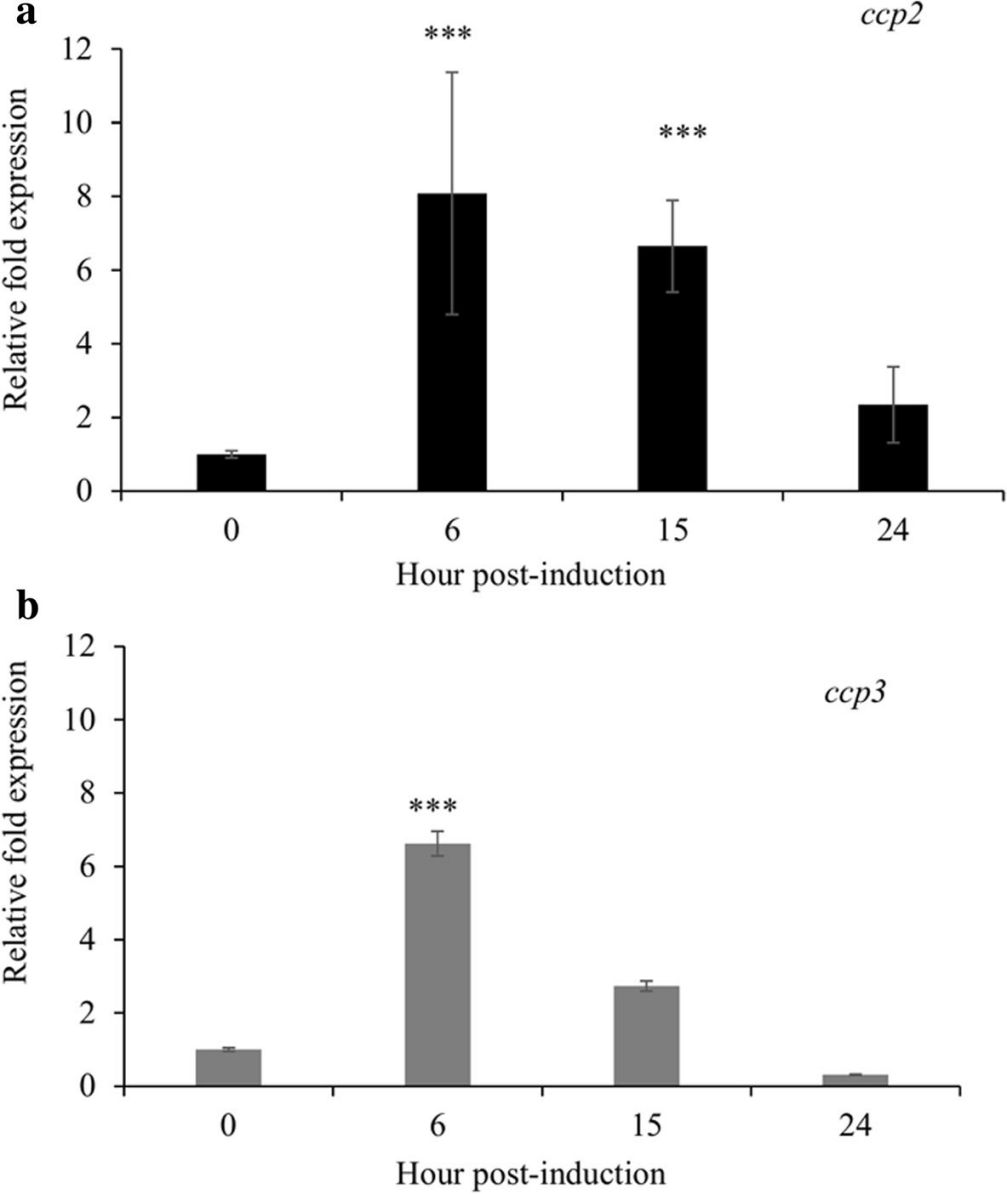
Relative expression of *B. bigemina ccp2* (**a**) and *ccp3* (**b**) by TCEP-induced sexual stages is compared with non-induced *B. bigemina* blood stages. The means of three experiments, each containing three technical replicates are shown. ***P < 0.001

**Fig. 4:**
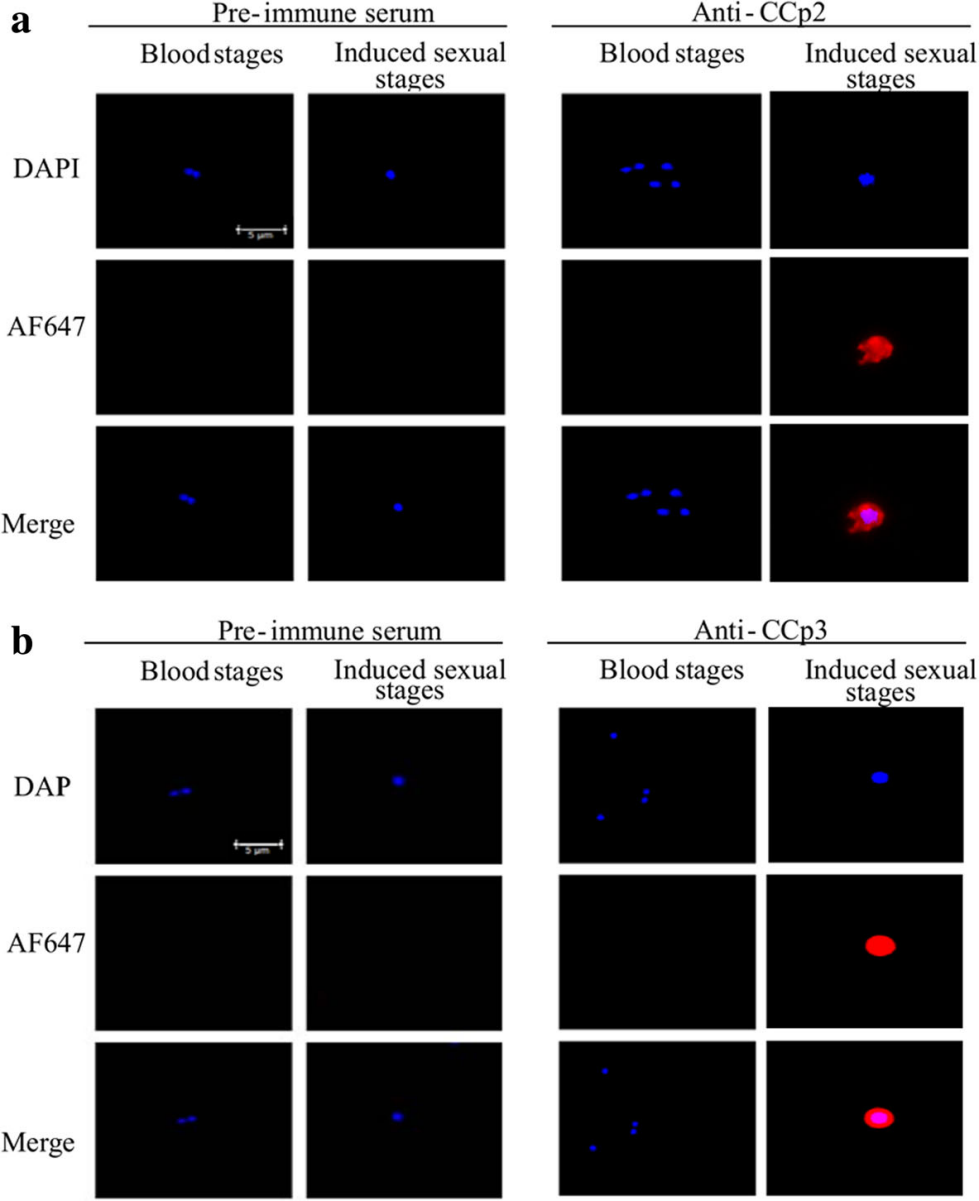
Immunofluorescence assays demonstrating the expression of CCp 2 (**a**) and CCp3 (**b**) in *in vitro* TCEP-induced *B. bigemina* sexual stages. *Scale-bars*: 5 μm

We compared the transcription levels of *B. bigemina* sexual stage marker genes *ccp2* and *ccp3* between cultures induced with 20 mM TCEP at 37 °C in 5% CO_2_ to non-induced cultures. Expression of *ccp2* and *ccp3* was found to be significantly upregulated in TCEP-induced cultures (*P* < 0.001) as compared to time zero (non-induced) parasites. The expression of *ccp2* was maximal by 6 h and gradually reduced over subsequent time points (Fig. 3a). *ccp3* expression was also maximal in 6 h cultures but experienced a sharp decline after 15 h of cultures (Fig. 3b). The results represent the means of three experiments, each containing three technical replicates. In addition to the *ccp* genes, we examined the expression of *B bigemina hap2* (BBBOND_0307410) in TCEP-induced parasites and found it was upregulated at 6, 15 and 24 h (Additional file 2: Figure S2) as previously demonstrated [13]. Additionally, we confirmed polypeptide expression of CCp2 and CCp3 by IFA using a set of previously characterized polyclonal antibodies [12]. Consistent with a previous study of induced *B. bigemina* sexual stages [12], the results demonstrated that *in vitro* TCEP-induced parasites expressed both CCp2 and CCp3 proteins but non-induced blood stages did not express either protein (Fig. 4a, b).

### *In silico* identification of *B. bigemina* sexual stage genes

*In silico* bioinformatic analysis was performed to identify *B. bigemina* homologs of *P. falciparum* gamete-specific genes. Using this approach, 13 *B. bigemina* homologs of *P. falciparum* gamete-specific genes were identified that contained conserved domains such as methyltransferase, tubulin-tyrosine ligase (TTL), kinesin, ribosomal _L20, tim10/DDP family zinc finger (zf-Tim10_DDP), 2-C-methyl-D-erythritol 4-phosphate cytidylyltransferase (IspD), U1 zinc finger (zf-U1), sedlin, N-terminal conserved region (Sedlin_N), RNA polymerases N/8 kDa subunit (RNA_pol_N) and zinc finger C-x8-C-x5-C-x3-H type (ZnF_C3H1) and LCCL domain with *P. falciparum* [23]. The amino acid identities of these homologs ranged between 18–58%. Accession numbers, amino acid identities and functional annotation for *B. bigemina* homologs of *P. falciparum* gamete-specific proteins are provided in Table 3.

**Table 3:** Protein identity between *B. bigemina* homologs of *P. falciparum* gamete-specific proteins

Protein name	*B. bigemina* accession number	*P. falciparum* accession number	Identity (%)	Function annotation
BBBOND_0204030	XP_012767431.1	XP_001348700.2	31.63	Hypothetical protein conserved
BBBOND_0310420	XP_012769325.1	XP_002808689.1	23.97	Tubulin-tyrosine ligase family protein
BBBOND_0110620	XP_012766950.1	XP_001348145.2	27.91	Kinesin putative
BBBOND_0308930	XP_012769176.1	XP_001348883.1	52.07	Ribosomal protein L20, putative
BBBOND_0200260	XP_012767055.1	XP_001351587.1	48.75	Mitochondrial import inner membrane translocase subunit Tim13 partial mRNA
BBBOND_0311670	XP_012769450.1	XP_002808921.1	18.25	2-C-methyl-D-erythritol 4-phosphate cytidylyltransferase
BBBOND_0208380	XP_012767870.1	XP_001351380.1	27.94	Hypothetical protein, conserved
BBBOND_0305250	XP_012768808.1	XP_001349398.1	38.21	U1 zinc finger family protein, putative
BBBOND_0307580	XP_012769040.1	XP_001350054.1	37.04	Sedlin, N-terminal conserved region family protein, putative
BBBOND_0310760	XP_012769359.1	XP_001348995.1	57.97	RNA polymerases N / 8 kDa subunit family protein, putative
BBBOND_0311270	XP_012769410.1	XP_001351786.1	31.47	Zinc finger C-x8-C-x5-C-x3-H type domain containing protein, putative
BBBOND_0203950	XP_012767423.1	XP_001615829	31.74	LCCL domain-containing protein CCP2, putative
BBBOND_0312400	XP_012769523.1	XP_001348240	36.83	LCCL domain containing protein, putative

### Expression of BBBOND_0204030 in *in vitro* TCEP-induced *B. bigemina* sexual stages

**Fig. 5:**
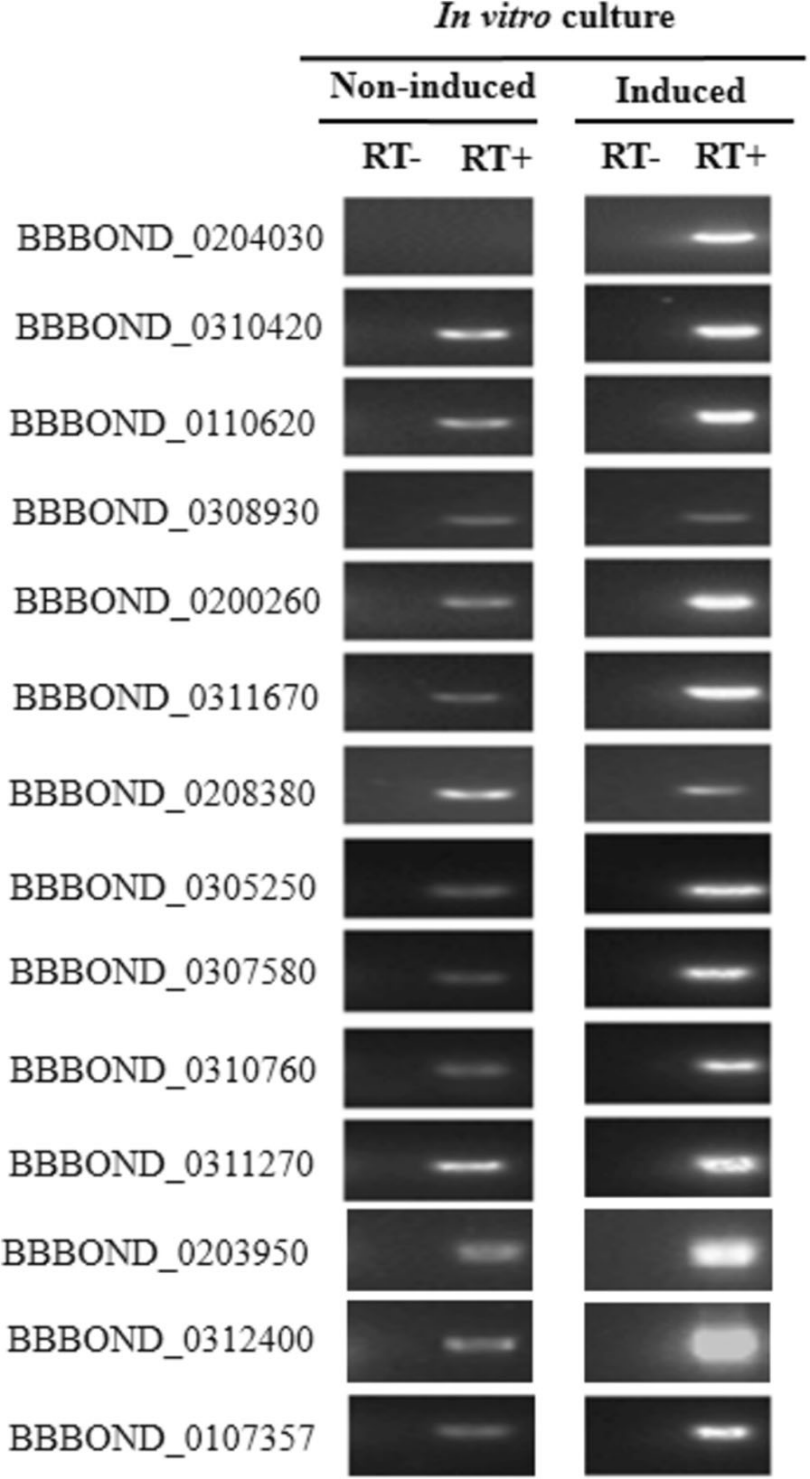
Detection of *B. bigemina* homologs of *P. falciparum* gamete-specific gene transcripts in *in vitro* TCEP-induced and non-induced blood cultures. BBBOND_0107357 (actin) was used as a reference gene. RT- and RT+ indicate the absence or presence of reverse transcriptase

Comparative RT-PCR analysis using total RNA isolated from *B. bigemina* non-induced and *in vitro* TCEP-induced cultures was performed to determine whether stage-specific markers can be identified among the 13 candidate *B. bigemina* homologs to conserved *P*. *falciparum* sexual stage genes. Expression of several *B. bigemina* homologs were detected in non-induced and induced cultures (Fig. 5); however, expression of BBBOND_0204030 was exclusive to induced cultures. This observation was confirmed by nested PCR analysis (Additional file 3: Figure S3). PCR products for all genes were sequenced, and amplicons were found to be more than 98% identical with the reference *B. bigemina* genome [27]. The newly generated sequences were submitted to the GenBank database under the accession numbers MH536857-MH536870.

### BBBOND_0204030 is uniquely expressed *in vivo* by *B. bigemina* tick stages

**Fig. 6:**
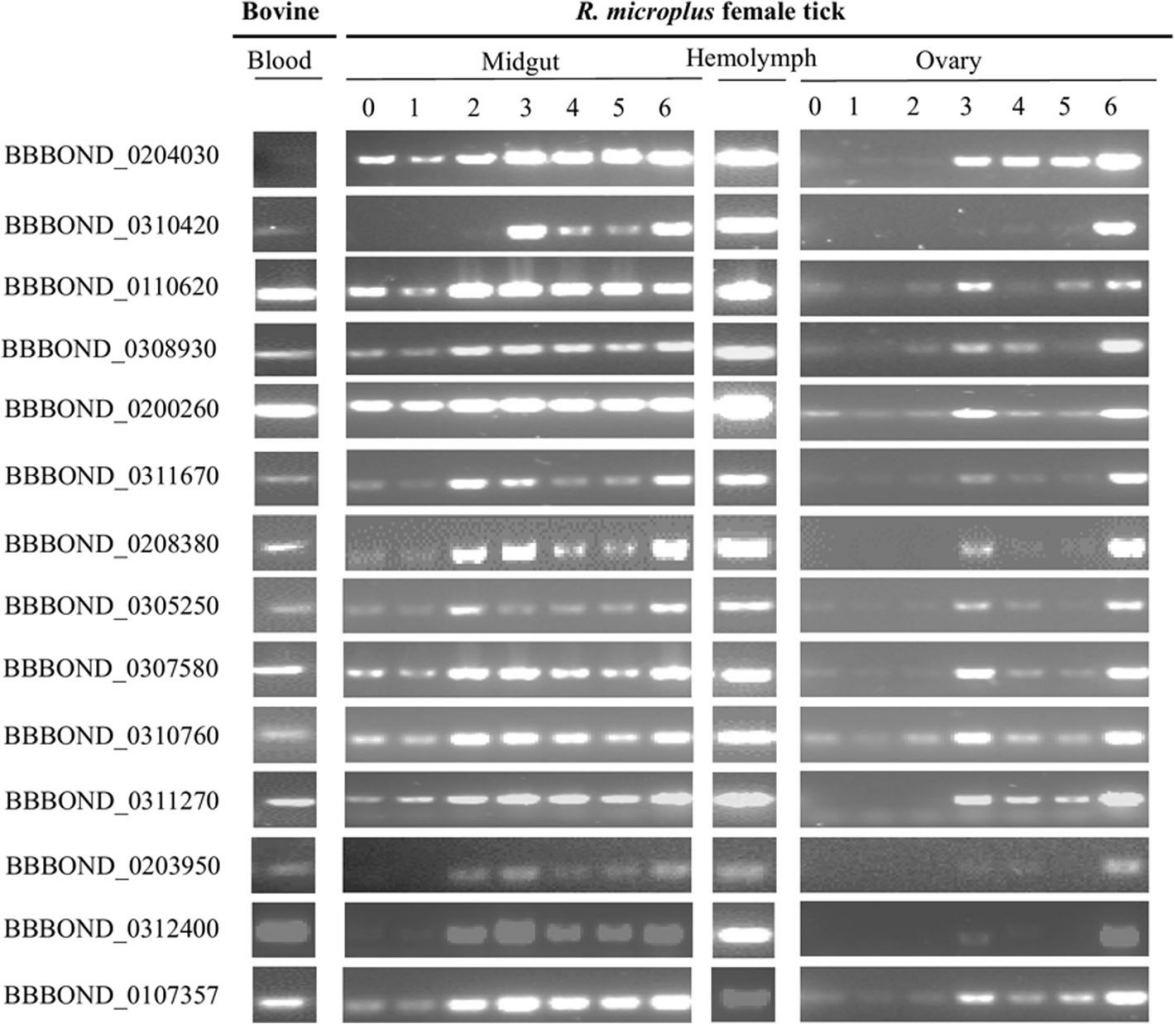
Detection stock of *B. bigemina* homologs of *P. falciparum* gamete-specific gene transcripts. Blood, midguts day 0 to 6 post-incubation of engorged female ticks, hemolymph, ovaries day 0 to 6 post-incubation of engorged female ticks indicated above lanes. BBOND_0107357 (actin) was used as a reference gene.

Gene expression results of the selected *B. bigemina* tick stage candidate genes demonstrated that 12 of 13 genes were transcribed in both *B. bigemina* blood and tick stages at various time-points after incubation in gut, ovary, and hemolymph samples. As already stated, gene BBBOND_0204030 was not transcribed by blood stages but was detected in all stages of *B. bigemina* infected tick gut, ovary and hemolymph (Fig. 6). These results were consistent with the *in vitro* observation described above. The PCR products were sequenced, and the amplicon identities confirmed.

## Discussion

In this study, we used *B. bigemina* sexual stages induced *in vitro* and identified BBBOND_0204030, a putative methyl-transferase, as a marker of tick-stage development. Methyl transferases are membrane-bound enzymes in the endoplasmic reticulum that transfer a methyl group from S-adenosyl-l-methionine to thiol, amino, or catechol acceptor molecules [35]. Protein methyltransferases are essential for epigenetic regulation of gene expression through methylation of histones and non-histone proteins such as transcription factors. In malaria, the homologous gene, PF14_0526, contains a methyltransferase domain that plays a fundamental role in parasite development and differentiation [35]. We found that BBBOND_0204030 was transcribed by multiple stages of *B. bigemina* during parasite development in tick midgut, ovary and hemolymph. Given the expression of BBBOND_0204030 in each tick compartment examined, the protein may play a central role in regulating genes essential for the parasites survival and development in the tick. BBBOND_0204030 transcripts were not found in blood stages of *B. bigemina*. To the best of our knowledge, there is no description of a *B. bigemina* gene transcribed exclusively during tick infection. We propose that BBBOND_0204030 can be used as a marker to indicate life-cycle transition of *B. bigemina* within tick vectors.

The dissection and characterization of mechanisms leading to gamete formation are difficult to perform in the complex milieu of the engorged female tick midgut. Thus, using TCEP to induce *B. bigemina* sexual stages *in vitro* will facilitate the study of gene and protein expression contributing to the understanding of molecular mechanisms involved in the parasite life-cycle and the development of novel transmission blocking vaccines. Examining previously defined sexual stage-specific *B. bigemina* genes *ccp2* and *ccp3*, we confirmed sexual stage formation by detecting upregulated transcription levels and protein expression by RT-qPCR and IFA, respectively [12]. When sexual stages were induced *in vitro*, *ccp2* and *ccp3* displayed a distinct expression pattern that included a 6–8-fold increase within 6 h of induction and subsequent decline. In contrast, the expression of both genes from *ex vivo* samples extended over the course of five days. It is possible that with *in vitro* induction, all parasites, simultaneously exposed to the induction stimulus, respond as a synchronous group, allowing the expression pattern to be revealed. In contrast, parasites in the tick are exposed over a long feeding period. As a result, *in vivo* induction of sexual stages would be expected to occur non-synchronously and the rise and fall of *ccp* expression would be masked. In addition to *ccp2* and *ccp3*, expression of *hap2*, a gene found to be important in *B. bovis* sexual stage development [16], was upregulated at 6 h but, unlike the expression pattern of the *ccp* genes, remained elevated at 15 and 24 h after TCEP induction (Additional file 2: Figure S2), consistent with a previous study [13].

*Babesia bigemina* has a complex life-cycle that includes the development of multiple stages in the mammalian host and tick vector [1, 6]. The disparate environments provide stress to the parasites that can induce differential gene and protein expression. Within the biological vector, *Babesia* sexually reproduce, undergo several morphological changes, and interact with multiple cell types and tissues including tick midgut, ovaries, hemolymph and salivary glands before transmission to animals [1, 6]. In this study we used TCEP to consistently induce culture adapted *B. bigemina* sexual stages *in vitro* and found that CO_2_ was a critical parameter [32]. TCEP-induced cultures contained several apparent forms of *B. bigemina* sexual stages including parasites with long projections, round forms and clusters of round forms as previously described for sexual stages found in tick midgut [1, 6]. The shapes and forms were similar to previously described sexual stages induced *in vitro* from *ex vivo* blood by either xanthurenic acid or addition of homogenized engorged female *R. microplus* tick midgut [16, 19, 34].

Xanthurenic acid failed to induce sexual stages from our *B. bigemina* strain using *in vitro* culture source material. One possible explanation is that not all strains of *B. bigemina* respond to the mild stress caused by xanthurenic acid. TCEP and DTT are both reducing agents and may have the same function in triggering endoplasmic reticulum stress that causes activation of *Plasmodium* transcriptional factors [20, 21]. Studies have shown that disruption of *P. falciparum* metabolic pathways resulted in the accumulation of S-adenosyl-L-homocysteine in infected erythrocytes with an upregulation of parasite transcriptional factors important in gametogenesis [20, 21].

Due to the consistent ability of TCEP to induce *B. bigemina* from our *in vitro* adapted cultures, this induction system represents an alternative gametogenesis technique for *B. bigemina* strains that fail to respond to xanthurenic acid treatment. We were able to detect morphological and transcriptional changes 6 h post-exposure with various concentrations of TCEP in the presence of CO_2_. The addition of xanthurenic acid did not impact TCEP induction of *B. bigemina* sexual stages in our experiments. Xanthurenic acid was previously identified in mosquito guts as an agent capable of inducing sexual stage differentiation of the *Plasmodium* parasite [36, 37]. However, it remains unknown whether xanthurenic acid is present and/or plays a similar role in the tick vectors of *B. bigemina*. It is likely that there is a distinct midgut milieu composition between *Anopheles* mosquitoes and *Rhiphicephalus* ticks.

To further characterize TCEP-induced *B. bigemina* sexual stages, we searched for homologous malaria gametogenesis gene markers. *In silico* analysis identified thirteen *B. bigemina* homologs of *P. falciparum* gamete-specific genes. From these 13 identified genes, 12 were transcribed in both non-induced and TCEP-induced *in vitro* cultures. This observation may indicate the presence of developmentally arrested sexual forms in the blood, similar to malaria [38]. To demonstrate if the 13 *B. bigemina* genes shared an expression profile between both *in vitro* and *in vivo* conditions, we also analyzed the pattern of expression of these genes in blood from a splenectomized calf experimentally infected with *B. bigemina* and in different tissues from acquisition fed ticks. Importantly, transcript analysis of *B. bigemina* infected blood and multiple tick organs demonstrated an identical expression profile of these 13 genes as compared to both non-induced and *in vitro* TCEP-induced cultures. There appeared to be differences in transcription levels of the 12 genes not uniquely expressed by tick stages between the different stages we examined. Further characterization of the magnitude of these differences will require additional RT-qPCR studies that are not within the scope of this report.

## Conclusions

In this study, we identified BBBOND_0204030 as a marker for parasite development within the tick vector. Additionally, we describe the use of TCEP to consistently induce *B. bigemina* sexual stages *in vitro*. TCEP *in vitro* induction will fundamentally enhance the study of gene and protein expression during *B. bigemina* sexual stage development with the potential to impact the control of parasite transmission. *In vitro* induction using TCEP of *B. bigemina* sexual stages and the discovery of a tick stage-specific gene marker, together with our recent ability to manipulate the genome of *B. bigemina,* will expand the toolbox available for closing important research gaps regarding parasite-tick interactions that limit the development of novel control strategies against these devastating tick-borne parasites of cattle.

## Additional files

Additional file 1: **Figure S1.** *Babesia bigemina* non-induced and TCEP-induced parasites viability were determined with 6-CFDA. Non-induced blood culture (**a**) and *in vitro* TCEP-induced sexual stages (**b**) at 37 °C and 5% CO_2_. *Scale-bar*: 5 μm. (TIF 267 kb)

Additional file 2: **Figure S2.** Relative expression of *Babesia bigemina hap2* by TCEP-induced sexual stages is compared with non-induced *B. bigemina* blood stages. The means of three experiments, each containing three technical replicates are shown. *P < 0.001. (TIF 98 kb)

Additional file 3: **Figure S3.** Detection methyltransferase gene (BBBOND_0204030) transcript of *Babesia bigemina* in *in vitro* TCEP-induced cultures using nested PCR. BBBOND_0107357 (actin) was used as a reference gene. RT- and RT+: indicate the absence or presence of reverse transcriptase. (TIF 145 kb)

## Abbreviations

DTT: dithiothreitol
HMDS: hexamethyldisilizane
PBS: phosphate-buffered saline
TCEP: Tris 2-carboxyethyl phosphine
