# Supplementary figures and images for: Identification of a putative methyltransferase gene of Babesia bigemina as a novel molecular biomarker uniquely expressed in parasite tick stages
Source: Parasit Vectors. 2018 Aug 24;11:480. doi: 10.1186/s13071-018-3052-9 (PMC6109354; doi:10.1186/s13071-018-3052-9)

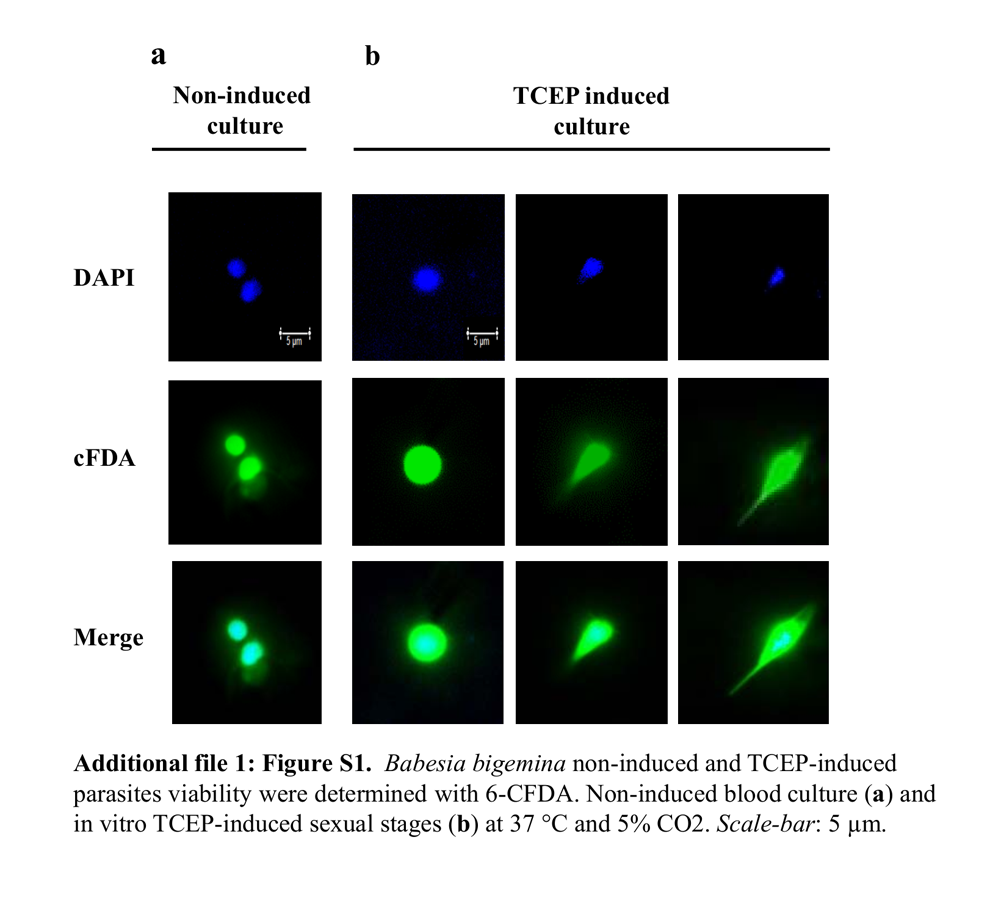

Supplement: Supplementary file 1 — Figure S1. Babesia bigemina non-induced and TCEP-induced parasites viability were determined with 6-CFDA. Non-induced blood culture (a) and in vitro TCEP-induced sexual stages (b) at 37 °C and 5% CO2. Scale-bar: 5 μm. (TIF 267 kb) [file 13071_2018_3052_MOESM1_ESM.tif]

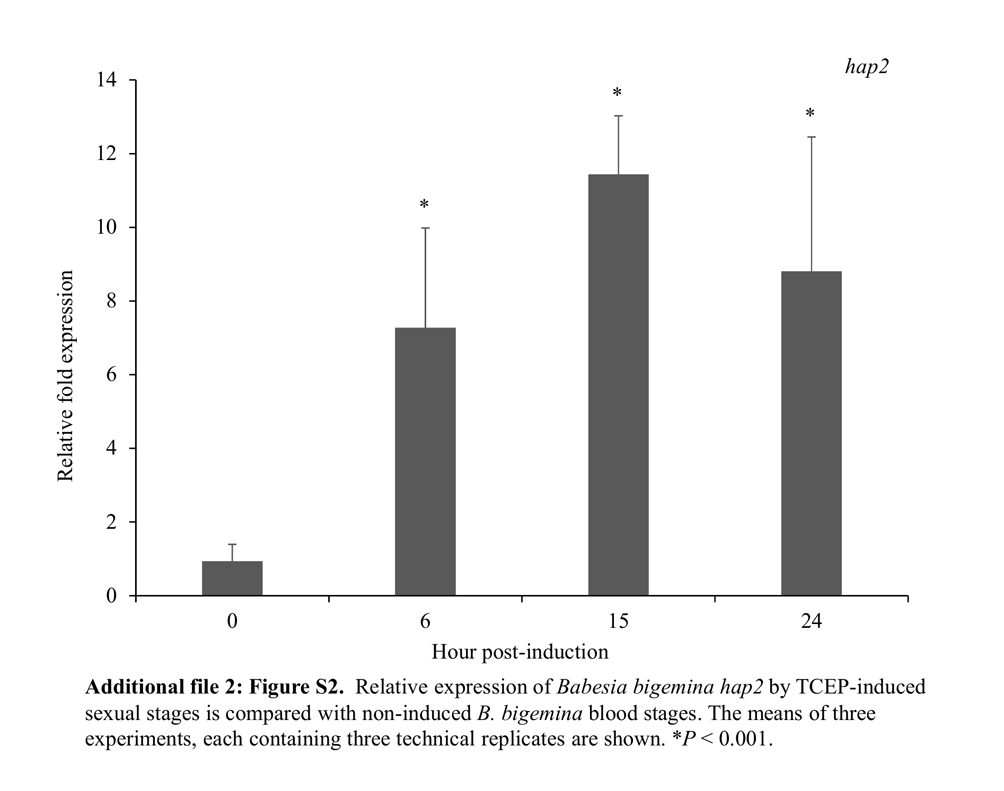

Supplement: Supplementary file 2 — Figure S2. Relative expression of Babesia bigemina hap2 by TCEP-induced sexual stages is compared with non-induced B. bigemina blood stages. The means of three experiments, each containing three technical replicates are shown. *P < 0.001. (TIF 98 kb) [file 13071_2018_3052_MOESM2_ESM.tif]

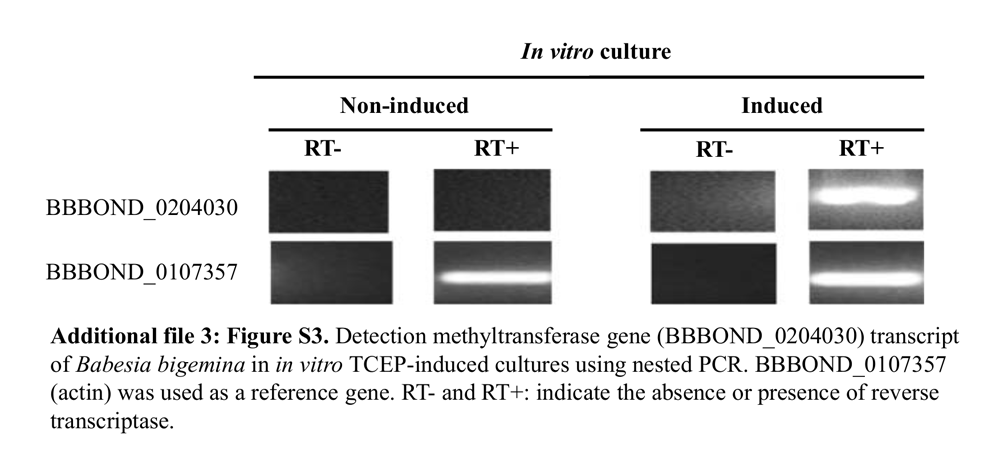

Supplement: Supplementary file 3 — Figure S3. Detection methyltransferase gene (BBBOND_0204030) transcript of Babesia bigemina in in vitro TCEP-induced cultures using nested PCR. BBBOND_0107357 (actin) was used as a reference gene. RT- and RT+: indicate the absence or presence of reverse transcriptase. (TIF 145 kb) [file 13071_2018_3052_MOESM3_ESM.tif]
